# The Role of Compassionate and Self-Image Goals in Predicting Psychological Controlling and Facilitative Parenting Styles

**DOI:** 10.3389/fpsyg.2019.01041

**Published:** 2019-06-05

**Authors:** James N. Kirby, Olivia Grzazek, Paul Gilbert

**Affiliations:** ^1^School of Psychology, The University of Queensland, Brisbane, QLD, Australia; ^2^College of Health and Social Care Research Centre, University of Derby, Derby, United Kingdom

**Keywords:** compassion, compassionate goals, parenting, motivation, compassion focused therapy

## Abstract

People enter into parental roles with a range of different motivations for parenting. To date, however, there is limited research assessing maternal motivations, concerns, and anxieties in their parenting styles. While some mothers are confident and child focused, others have concerns with performing parenting behaviors, and can be self-focused, shame prone, and self-judgmental. Two studies explored these two dimensions in relation to degree of controlling and facilitative parenting styles in the mothers of 3–9-year-old children. In study one, 151 mothers took part in an online survey measuring these two dimensions using the compassionate goals and self-image goals scales (Crocker and Canevello, 2008), in relation to facilitative and controlling parenting styles. As predicted, after controlling for child behavior, parental mental health, and parental self-efficacy, self-focused and shame avoidant concerns were associated with greater psychologically controlling parenting. In contrast a compassionate focused orientation was associated with greater facilitative parenting. In study two, 198 mothers were randomly assigned to either compassion focused goals, self-image goals, or control condition, which was manipulated by varying the instructions provided to participants. Emotional responses (e.g., angry, sad, and shame) to difficult parenting scenarios did not differ depending on whether participants were prompted with compassionate goal, self-image goal, or control condition instructions. The findings from study 1 demonstrate how goal motivation can influence parenting style, with the results from study 2 suggesting that instruction alone is insufficient to shift goal orientation.

## Introduction

Parenting style is linked to a range of maturational processes in the child including: on epigenetics (Cowan et al., 2016), brain development (Belsky and de Haan, 2011), attachment (Mikulincer and Shaver, 2016), emotional responding (Eisenberg et al., 1991), self-control (Cecil et al., 2012), and social and communicative competence (Hart et al., 2003). A number of factors influence parenting including, the ecological and social environment (e.g., poverty) (Perkins et al., 2013); stress (Anthony et al., 2005); mental health (Rodgers, 1998); parental knowledge and competency (Sanders and Mazzucchelli, 2018); self-efficacy (Sanders and Woolley, 2005), and the nature, disruptiveness and severity of child behavior problems (Jackson, 2000). Over the last 30 years various parenting programs have been developed to target parental factors of skills and competency to improve outcomes for children (Sanders and Kirby, 2014; Kirby, 2016).

A number of authors have also highlighted the importance of assessing parental motives and concerns as sources of variation in parenting (Abidin, 1992; Sanders and Mazzucchelli, 2018; Kirby et al., 2019). Indeed there are a number of different motives underpinning maternal parental styles including: authoritarian versus authoritative (Robinson et al., 1995); facilitative, which is work around and with the child’s needs, in contrast to regulating, which seeks to enable the child to fit into routines and structures of the family and parent (Raphael-Leff, 1986). Koren-Karie et al. (2002) explored three types of maternal interaction: (1) *positively insightful mothers* who tried to orientate their behaviors by trying to see the world through the child eyes; in contrast (2) *one-sided mothers* who had clear ideas about what the child needed and how the child should act, they tended to impose care; and (3) *disengaged mothers* who struggled to relate to their children. One crucial dimension to parental motives and concern is the degree to which parents feel confident in their parental role in contrast to uncertain, self judgmental, shame prone, and shame avoidant.

### Compassionate and Self-Image Goals

Crocker and her colleagues (Crocker and Canevello, 2008; Crocker et al., 2009) developed a measure to tap into these dimensions of social relating: labeled compassionate goals (i.e., desires to be helpful) and self-image goals (i.e., concerns with doing things wrong and being rejected). Crocker et al. (2009) theorized that people with self-image goals typically view relationships with others from an *egosystem* motivational perspective by prioritizing their own anxieties and needs at the expense of others. People adopt self-image goals to construct, maintain and defend a public image that reflects their ideal self (Crocker and Canevello, 2008; Crocker et al., 2009). Self-image goals are self-focused, defensive and typically adopted by those lacking in social confidence as a safety behavior to avoid rejection, which paradoxically leads to decreased regard from others, and decreased self-esteem, and less secure relating (Canevello and Crocker, 2011). Self-image goals tend to be associated with high emotional arousal such as shame, anger, and sadness (Crocker and Canevello, 2011).

In contrast, compassionate goals are other-focused and operate from an *ecosystem* motivational perspective (Crocker et al., 2009). When operating with compassionate goals, people want to be helpful to, and avoid harming, others (Crocker and Canevello, 2008). Compassionate goals are associated with increased self-esteem and regard from others (Canevello and Crocker, 2011), and foster positive emotions such as feeling at ease and connected to others (Canevello and Crocker, 2017).

There is accumulating evidence showing that compassionate and self-image goals reflect distinct motivational perspectives (Crocker and Canevello, 2008; Canevello and Crocker, 2011; Erickson et al., 2018). Crocker and Canevello (2008) examine intrapersonal effects of goals on perceived social support and trust in 199 students. Those with high levels of compassionate goals and low self-image goals reported greater perceived social support and trust, and reduced conflict. Conversely, self-image goals were associated social anxiety, defensive beliefs, and increased conflict and loneliness. Compassionate and self-image goals have not been specifically examined in parents but these motives may play a role in tendencies to be controlling.

### Controlling vs. Facilitative Parenting Styles

Parenting that is highly controlling is linked to poor child outcomes such as anxiety (Laurin et al., 2015) and lower social competence (McDowell et al., 2003). Conversely, positive and facilitative parenting practices are linked to positive child outcomes, including increased social competence (McDowell et al., 2003) and reduced likelihood that a child will develop antisocial behaviors, despite the influence of neighborhood deprivation, poverty and low socio-economic status (Odgers et al., 2012).

Controlling parenting is a highly intrusive form of parenting, whereby the parent attempts to control the child’s thoughts, self-expression, feelings, and attachment to the parent (Barber, 1996; Barber and Harmon, 2002). Psychological control strategies include inducing guilt and anxiety, and withdrawing love, in order to control the child (Barber and Harmon, 2002). Mills et al. (2007) examined the link between shame and psychological control among 198 mother-father pairs of children aged 3.6 to 4.5 years. A negative approach to the child mediated the association between shame and critical/rejecting parenting. The authors suggested that shame prone parents may project shame onto their child, leading to negative feelings that increase critical/rejecting behavior. Proneness to shame, characterized by self-focused concerns, may be an important factor that leads to psychological control (Mills et al., 2007). Given this, self-image goal orientation, which are significantly correlated with shame (Crocker and Canevello, 2011) and concern with others’ judgments of self (Canevello and Crocker, 2011), may also be significantly associated with controlling parenting.

In contrast, facilitative parenting promotes independence, rather than being overly directed or protected (Healy et al., 2015b). Facilitative parenting encompasses warm and responsive parenting behaviors that support the child’s social skills and peer relationships (Healy et al., 2015b). It involves coaching social and emotion regulation skills, managing parent-child conflict and encouraging socialization with peers (Healy et al., 2015a). Facilitative parenting has been linked to positive social, emotional, and behavioral outcomes (Healy et al., 2015a). Miller et al. (2015) explored the relationship between compassion, autonomic nervous system activity, and parenting behaviors among 83 mothers during challenging interactions with their child. Mothers and their 3.5-year-old child were provided with two tasks. During the difficult puzzle task, mothers could give as much assistance to the child as required. During an origami task, mothers were instructed to provide verbal instruction only. Miller et al. (2015). found that greater self-reported compassion for one’s child was associated with greater observed warmth, reduced observed negativity, and reduced harsh parenting. Miller et al. (2015) utilized observational and physiological measures, supporting the conclusion that a compassion orientation protected against adverse parenting practices, even among those who experienced strong physiological stress. To date, however, there has been no direct exploration of compassionate and self-image goals in relation to controlling and facilitative parenting. Examining these links could provide insights into how motives influence parenting style, and thus offer a modifiable target (e.g., parent motivation) in parenting programs to help improve parent-child relating and positive childhood social, emotional and behavior development.

### The Current Research

Self-image and compassionate goals shape relationships with others (Crocker and Canevello, 2008; Canevello and Crocker, 2011; Erickson et al., 2018).

The aim of the first study was to examine the relationship between (a) compassionate goals, and (b) self-image goals, and facilitative and psychologically controlling parenting styles.

Study 2 sought to extend on previous research by examining the impact of priming mothers with different goal orientations, and exploring their emotional responses to difficult mother-child interactions.

## Study 1

The first study involved a cross-sectional survey design to examine whether compassionate and self-image goals in parents explain variance in their implementation of facilitative and psychologically controlling parenting behaviors. Participants completed an online questionnaire about their child’s behavior, parental mental health, self-efficacy, interpersonal goals, and parenting. Child behavior, parental mental health, and self-efficacy were included as they are known to influence parenting behavior (Mash and Johnston, 1990; Abidin, 1992; Rodgers, 1998; Jackson, 2000; Anthony et al., 2005; Sanders and Woolley, 2005). To measure compassionate and self-image goals in parents, the Compassionate and Self-Image Goals Scale, developed by Crocker and Canevello (2008), Study 2, was adapted to apply to a parenting context.

Based on the theory that self-image goals involve prioritizing one’s own needs at the expense of others (Crocker et al., 2009), it was hypothesized that self-image goals would positively predict psychologically controlling parenting, over and above variance explained by child behavior, parental mental health, and parental self-efficacy (H1). In contrast, given that compassionate goals are associated with responsiveness to other’s needs (Canevello and Crocker, 2011) and belief in interconnectedness with others (Crocker and Canevello, 2008), it was hypothesized that compassionate goals would positively predict facilitative parenting, over and above variance explained by child behavior, parental mental health, and parental self-efficacy (H2). We also explored whether self-image goals would explain variance in facilitative parenting, and whether compassionate goals would explain variance in psychological control, over and above child behavior, parental mental health, and self-efficacy.

## Materials and Methods

Both Study 1 and 2 were granted ethical approval by the University of Queensland ethics review committee in accordance with the National Health and Medical Research Council’s guidelines (clearance number: 18-PSYCH-4-71-JMC). Both studies were preregistered with the Open Science Framework^1^.

### Design and Participants

The study was a cross-sectional survey design. Predictor variables were child behavior, parental mental health, self-efficacy, and compassionate and self-image goals. Based on past research, the parent’s age and the age of their child were included as control variables (Sanders et al., 2014). Outcome variables were psychological control and facilitative parenting. An a priori power analysis using the software program G^∗^Power (Faul et al., 2007) indicated that 103 participants would be required to obtain adequate power (0.80) to detect a medium *r* effect of 0.15 at the standard 0.05 alpha error probability.

Two-hundred and nineteen respondents voluntarily accessed the survey. Parents with a child aged 3–9 years were eligible to participate. Forty-four participants were excluded (child not aged 3–9 years, *n* = 8; missing all data, *n* = 28; only provided demographic information, *n* = 8). There were 11 males (6.29%) and 164 females (93.71%). The majority of participants in previous parenting research have been mothers (Nowak and Heinrichs, 2008) and there was a small proportion of males in the current study, thus, the decision was made to remove males from further analyses. A further 13 participants were excluded due to inadequate sampling. Inadequate sampling refers to the participants that were excluded due to missing a large proportion of data. Thirteen participants were missing over 77.50% of the data points and were therefore deemed inadequately sampled. The final sample consisted of *N* = 151 mothers aged 19–55 years (*M* = 35.24 years, *SD* = 6.14), with at least one child aged 3–9 years (*M* = 5.45 years, *SD* = 1.94). Additional participant demographic information is provided in Table 1.

**Table 1:** Study 1: participant demographic characteristics (*N* = 151).

Characteristics	*N*	%
**Education**		
Some high school	8	5.30
Completed high school	13	8.60
Tertiary or tafe course	73	48.30
Postgraduate degree	57	37.70
**Employment status**		
Full-time	37	24.50
Part-time	55	36.40
Casual	14	9.30
Employed, but on maternity leave	11	7.30
Full-time student	6	4.00
Unemployed, looking for work	3	2.00
Not in paid employment	25	16.60
**Income (*n* = 148)**		
0 – 10,000	23	15.20
$10,001 – 20,000	7	4.60
$20,001 – 30,000	14	9.30
$30,001 – 40,000	22	14.60
$40,001 – 50,000	14	9.30
$50,001 – 60,000	24	15.90
$60,001 – 70,000	9	6.00
$70,001 – 80,000	7	4.60
$80,001 – 90,000	7	4.60
$90,001 – 100,000	5	3.30
$100,001+	16	10.60
**Ethnicity (*n* = 150)**		
Caucasian Australian	131	86.80
Pacific Islander	1	0.70
Asian	4	2.60
Aboriginal/Torres Strait Islander	1	0.70
Other	13	8.60
**Relationship status**		
Single	11	7.30
Married/defacto	132	87.40
Separated/Divorced	8	5.30
**Household dynamic**		
Original family (both biological or adoptive parents present)	121	80.10
Step-family (two parents, one being a step-parent)	7	4.60
Sole parent family	16	10.60
Other	7	4.60
**Number of children**		
One	25	16.60
Two	85	56.30
Three	29	19.20
Four	8	5.30
Five	4	2.60
**Social, emotional, behavioral concerns with child**		
Yes	63	41.70
No	88	58.30

*N = 151 (unless otherwise specified).*

### Measurements

#### Demographic Information

The Family Background Questionnaire (FBQ; Zubrick et al., 1995) was used to collect demographic information, as reported in Table 1.

#### Child Behavior

The Strengths and Difficulties Questionnaire (SDQ; Goodman, 1997) consists of 25 items measuring parents’ perceptions of their child’s prosocial and difficult behaviors. There are five subscales each containing five items, measuring emotional symptoms, conduct problems, hyperactivity/inattention, peer relationship problems, and prosocial behavior. Participants responded on a three-point Likert scale from 0 (*Not True*) to 2 (*Certainly True*). The Total Difficulties score is calculated by summing the scores from all of the scales except the Prosocial Behavior scale, with higher scores indicating more problematic child behavior. We only examined Total Difficulties for the present study. The Total Difficulties score has previously shown good concurrent validity (Goodman, 1997). In the current study, the total score showed good internal consistency (α = 0.84).

#### Parental Mental Health

The Depression and Anxiety Stress Scale-21 (DASS-21; Lovibond and Lovibond, 1995) consists of three subscales measuring depression, anxiety, and stress. Participants indicated how much each item applied to them over the past week on a four-point Likert scale from 0 (*Did not apply to me at all*) to 3 (*Applied to me very much or most of the time*). A total score is calculated as a measure of parental mental health by summing all items. Higher scores indicate greater severity of symptoms. Based on a recent analysis of the psychometric properties of the DASS-21, we discarded item 5 when calculating the total score, and converted scored from ordinal level to interval level (Medvedev et al., 2019). The DASS-21 has previously demonstrated good convergent and discriminant validity (Henry and Crawford, 2005) and good internal consistency for the depression (α = 0.94), anxiety (α = 0.87), and stress (α = 0.91) subscales (Antony et al., 1998). In the current study, the total score showed excellent internal consistency (α = 0.92).

#### Parental Self-Efficacy

The parenting sense of competence scale (PSOC; Johnston and Mash, 1989) consists of 16 items that measure two dimensions of parental self-esteem, specifically, satisfaction and efficacy. Participants respond on a six-point Likert scale from 1 (*Strongly Agree*) to 6 (*Strongly Disagree*). For this study, a total score was calculated for the efficacy subscale. Scores are summed, with higher scores indicating higher parental self-efficacy. The PSOC has previously demonstrated convergent and divergent validity (Ohan et al., 2000) and acceptable internal consistency for the efficacy subscale (α = 0.76; Johnston and Mash, 1989). In the current study, internal consistency for the efficacy subscale was good (α = 0.81).

#### Compassionate and Self-Image Goals

The Compassionate and Self-Image Goals Scale (Crocker and Canevello, 2008, Study 2) consists of 16 items measuring interpersonal goals. The scale was adapted to apply to a parent-child context for the purpose of this study. The measure began with the phrase *“In the past week, in my relationship with my child(ren), I wanted/tried to…,”* followed by seven items measuring self-image goals (e.g., *“Avoid showing my weaknesses”*) and nine items measuring compassionate goals (e.g., *“Avoid being selfish or self-centered”*). Participants responded on a five-point Likert scale from 1 (*Not at all*) to 5 (*Extremely*). The mean for each of the compassionate and self-image subscales was calculated, with higher scores indicating higher interpersonal goals. The original subscales targeted at roommate relationships have demonstrated acceptable to excellent internal consistency (*M*_α_ = 0.95; self-image goals: *M_α_* = 0.83; Crocker and Canevello, 2008, Study 2). In the current study, internal consistency for each subscale was good (α = 0.84 for compassionate goals, 0.80 for self-image goals).

#### Psychological Control

The parental psychological control measure (PPC; Olsen et al., 2002) consists of 33 items that measures psychological control in terms of critical/rejecting parenting. There are six subscales, specifically, three items measuring constraining verbal expression, three items measuring invalidating feelings, three items measuring personal attacking, six items measuring erratic emotional behavior, five items measuring love withdrawal, and 13 items measuring guilt induction. Participants responded on a 5-point Likert scale from 1 (*Never*) to 5 (*Always*). For this study, a total score was calculated by summing responses, with higher scores indicating higher psychological control. In the current study, internal consistency for the PPC was excellent (α = 0.90).

#### Facilitative Parenting

The Facilitative Parenting Scale (FPS; Healy et al., 2015a) consists of 58 items measuring parental support for child friendships and peer skills. There are 11 subscales, specifically, Warmth, Supports Friendships, Not Over-Protective, Not Conflicting, Child Communicates to Parent, Parent Coaches, Communicates with Teacher, Not Over-Involved in School, Not Aggressively Defensive, Enables Independence, and Not Overly Directive. Participants responded on a five-point Likert scale from 1 (*Not true*) to 5 (*Extremely true*). There are 19 reverse-scored items. The mean score across all items was calculated, with higher scores indicating higher facilitative parenting. The scale has previously demonstrated good internal consistency (α = 0.89) and convergent validity (Healy et al., 2015a). In the current study, internal consistency was good (α = 0.86).

### Procedure

Participants completed the anonymous online questionnaire using Qualtrics^TM^ Survey Software. All the participants provided written online informed consent before the start of their participation. A convenience sample was used involving snowball sampling via online social media.

### Data Analysis Plan

Analyses for Study 1 and 2 were performed using IBM SPSS^TM^ software Version 25. Prior to conducting analyses, missing data, assumptions and descriptive statistics were examined. Bivariate correlations between variables were also assessed. Threshold for statistical significance for this study was α = 0.05, two-tailed.

A hierarchical multiple regression was conducted to test whether self-image goals explained variance in psychological control, over and above child behavior, parental mental health, and self-efficacy. A second hierarchical multiple regression was conducted to test whether compassionate goals explained variance in facilitative parenting, over and above child behavior, parental mental health, and self-efficacy. For both regression analyses, age of the mother and child in years were entered at Step One to control for demographics (Sanders et al., 2014). Child behavior (SDQ) was entered at Step Two and parental mental health (DASS-21) and self-efficacy (PSOC-Efficacy subscale) were entered at Step Three on a theoretical basis (Mash and Johnston, 1990; Abidin, 1992; Rodgers, 1998; Jackson, 2000; Anthony et al., 2005; Sanders and Woolley, 2005). Compassionate and self-image goals were entered simultaneously at Step Four to assess their unique effect while controlling for the other goal (Crocker and Canevello, 2008).

## Results

### Data Screening

#### Missing Data

Analyses were conducted to determine the pattern of missing data among key variables (SDQ, DASS-21, PSOC-Efficacy subscale, compassionate and self-image goals subscales, PPC, and the FPS). Missing Values Analysis revealed a non-significant little’s missing completely at random (MCAR) test χ^2^ (865, *N* = 151) = 824.23, *p* = 0.836, indicating that the data were MCAR. Expectation Maximization was used to estimate missing data at the item level.

### Preliminary Analyses

#### Descriptive Statistics

Descriptive statistics were calculated to obtain the means and standard deviations of the measures used in the study (see Table 2).

**Table 2:** Study 1: demographic characteristics, means and standard deviations for predictor and outcome variables.

Variable	Measure	Subscale	Mean	*SD*	Min.	Max.
**Predictor variables**						
Age of mother^a^	FBQ		35.24	6.14	19	55
Age of child	FBQ		5.45	1.94	3	9
						
Child behavior	SDQ		10.91	6.11	1.00	28.00
Parental mental health	DASS-21		11.35	8.57	0.00	51.00
Self-efficacy	PSOC	Efficacy	28.86	5.64	13.00	41.00
Goals	Compassionate and self-image goals scale	Compassionate goals	4.03	0.56	2.56	5.00
	Compassionate and self-image goals scale	Self-image goals	2.48	0.74	1.29	4.43
**Outcome variables**						
Psychological control	PPC		61.88	13.01	35.00	106.00
Facilitative parenting	FPS		3.77	0.33	2.64	4.45

*N = 151 (unless otherwise specified). ^a^n = 150 (Mother who did not provide age excluded from this analysis). FBQ, family background questionnaire; SDQ, strengths and difficulties questionnaire; DASS-21, depression and anxiety stress scale-21; PSOC, parenting sense of competence. Compassionate goals; Self-image goals; PPC, parental psychological control measure; FPS, facilitative parenting scale.*

#### Bivariate Relationships

As hypothesized, there was significant negative correlation between compassionate goals and psychological control (*r* = -0.41, *p* < 0.01), and a small-moderate positive correlation between compassionate goals and facilitative parenting (*r* = 0.32, *p* < 0.01). In relation to self-image goals, there was a small-moderate positive correlation with psychological control (*r* = 0.30, *p* < 0.01), and a small negative correlation between self-image goals and facilitative parenting (*r* = -0.27, *p* < 0.01). Interesting, there was a significant negative association between compassionate goals and total child difficulties (*r* = -0.19, *p* < 0.05), this was not found for self-image goals. All bivariate correlations are reported in Table 3.

**Table 3:** Study 1: bivariate correlations between key variables.

Variables	1	2	3	4	5	6	7	8	9
(1) Age of mother	1.00								
(2) Age of child	0.39^**^	1.00							
(3) Child behavior problems (SDQ-total score)	-0.21^**^	0.09	1.00						
(4) Mental health (DASS-21-total score)	-0.35^**^	0.01	0.35^**^	1.00					
(5) Parental efficacy (PSOC – efficacy)	-0.13	-0.06	-0.31^**^	-0.13	1.00				
(6) Compassionate goals	-0.12	-0.06	-0.19^*^	0.04	0.34^**^	1.00			
(7) Self-image goals	-0.29^**^	-0.02	0.12	0.17^*^	0.09	0.19^*^	1.00		
(8) Psychological controlling parenting (PPC)	0.03	0.13	0.35^**^	0.20^*^	-0.25^**^	-0.41^**^	0.30^**^	1.00	
(9) Facilitative parenting (FPS)	0.20^*^	0.04	-0.41^**^	-0.31^**^	0.34^**^	0.32^**^	-0.27^**^	-0.46^**^	1.00

*N = 150. SDQ, strengths and difficulties questionnaire; DASS-21, depression and anxiety stress scale-21; PSOC – Efficacy, parenting sense of competence – efficacy subscale; PPC, parental psychological control measure; FPS, facilitative parenting scale. ^∗^p < 0.05. ^∗∗^p < 0.01.*

### Main Analyses

#### Psychological Control

Results of the hierarchical multiple regression, see Table 4, revealed that mother’s and child’s age in years did not significantly contribute to the regression model at Step One, accounting for a non-significant 1.8% of variance in psychological control, Δ*R*^2^ = 0.018, Δ*F* (2, 147) = 1.37, *p* = 0.258. At Step 2, child behavior did contribute significantly, accounting for an additional 12.3% of variance in psychological control, Δ*R*^2^ = 0.123, Δ*F* (1, 146) = 20.95, *p* < 0.001, with greater child behavior problems being associated with greater psychological control (β = 0.37, *p* < 0.001). At Step 3, parental mental health and self-efficacy did not significantly contribute to the regression model, accounting for a non-significant 2.8% of variance in psychological control, Δ*R*^2^ = 0.028, Δ*F* (2, 144) = 2.43, *p* = 0.09. At Step 4, and as hypothesized compassionate and self-image goals contributed significantly to the regression model, and together accounted for an additional 23% of variance in psychological control, Δ*R*^2^ = 0.231, Δ*F* (2, 142) = 27.37, *p* < 0.001. As hypothesized, higher self-image goals were associated with greater psychological control, (β = 0.38, *p* < 0.001), accounting uniquely for 12.7% of variance. Higher compassionate goals was associated with lower psychological control (β = -0.41, *p* < 0.001), accounting for 14.1% of variance. Together, all seven predictor variables significantly accounted for 40.1% of variance in psychological control, *R*^2^ = 0.401, adjusted *R*^2^ = 0.371, *F* (7, 142) = 13.55, *p* < 0.001, indicating a large effect size, *f*^2^ = 0.67. In the final model the significant predictor variables were compassionate goals (14.1%), self-image goals (12.7%), and child behavior (3%).

**Table 4:** Study 1: summary of hierarchical regression analysis for variables predicting psychological control (PPC scores).

	B	*β*	95% CI	*t*	*sr^2^*	*R^2^*	*R^2^*(adj.)
**Step 1**						0.018	0.005
Age of mother (years)	-0.05	-0.02	[-0.42, 0.33]	-0.24	0.000		
Age of child (years)	0.96	0.14	[-0.22, 2.13]	1.60	0.017		
**Step 2**						0.141	0.124
Age of mother (years)	0.18	0.09	[-0.18, 0.54]	0.99	0.006		
Age of child (years)	0.46	0.07	[-0.67, 1.59]	0.80	0.004		
SDQ	0.78	0.37	[0.44, 1.12]	4.58^***^	0.123		
**Step 3**						0.169	0.141
Age of mother (years)	0.20	0.09	[-0.19, 0.59]	1.01	0.006		
Age of child (years)	0.14	0.06	[-0.71, 1.54]	0.73	0.003		
SDQ	0.61	0.29	[0.24, 0.98]	3.28^**^	0.062		
DASS-21	0.16	0.11	[-0.08, 0.41]	1.31	0.010		
PSOC-Efficacy	-0.31	-0.14	[-0.69, 0.06]	-1.64	0.015		
**Step 4**						0.401	0.371
Age of mother (years)	0.34	0.16	[-0.00, 0.68]	1.96	0.016		
Age of child (years)	0.24	0.04	[-0.73, 1.20]	0.48	0.001		
SDQ	0.43	0.20	[0.11, 0.75]	2.68^**^	0.030		
DASS-21	0.18	0.13	[-0.03, 0.39]	1.69	0.012		
PSOC-Efficacy	-0.11	-0.05	[-0.44, 0.23]	-0.61	0.002		
Compassionate goals	-9.53	-0.41	[-12.80, -6.27]	-5.77^***^	0.141		
Self-image goals	6.72	0.38	[4.30, 9.14]	5.49^***^	0.127		

*n = 150. SES, socio-economic status; CI, confidence interval; SDQ, strengths and difficulties questionnaire; DASS-21, depression and anxiety stress scale-21; PSOC – Efficacy, parenting sense of competence – efficacy subscale. ^∗^p < 0.05. ^∗∗^p < 0.01. ^∗∗∗^p < 0.001.*

#### Facilitative Parenting

Results of the hierarchical multiple regression, see Table 5, found mother’s and child’s age in years contributed significantly to the regression model at Step One, accounting for 4.3% of variance in facilitative parenting, Δ*R*^2^ = 0.043, Δ*F* (2, 147) = 3.28, *p* = 0.040. Age of the mother was the only significant predictor, accounting uniquely for 4.1% of variance, with older age associated with greater facilitative parenting (β = 0.22, *p* = 0.013). At Step 2, child behavior contributed significantly, accounting for an additional 13.6% of variance in facilitative parenting, Δ*R*^2^ = 0.136, Δ*F* (1, 146) = 24.21, *p* < 0.001, with greater child behavior problems being associated with lower facilitative parenting (β = -0.39, *p* < 0.001). At Step 3 parental mental health and self-efficacy contributed significantly, accounting for an additional 8.2% of variance in facilitative parenting, Δ*R*^2^ = 0.082, Δ*F* (2, 144) = 7.94, *p* < 0.001. Self-efficacy was the only significant predictor, accounting uniquely for 5.9% of variance, with higher self-efficacy being associated with greater facilitative parenting (β = 0.26, *p* < 0.001). At Step 4, compassionate goals and self-image goals contributed significantly accounting for an additional 11% of variance in facilitative parenting, Δ*R*^2^ = 0.110, Δ*F* (2, 142) = 12.35, *p* < 0.001. As hypothesized, higher compassionate goals were associated with higher facilitative parenting (β = 0.28, *p* < 0.001), accounting for 6.5% of unique variance, and higher self-image goals were associated with lower facilitative parenting (β = -0.27, *p* < 0.001), accounting for 6.3% of unique variance. Together, all seven predictor variables significantly accounted for 37% of variance in facilitative parenting, *R*^2^ = 0.370, adjusted *R*^2^ = 0.339, *F* (7, 142) = 11.91, *p* < 0.001, indicating a large effect size, *f*^2^ = 0.59. In the final model the significant predictors included compassionate goals (6.5%), self-image goals (6.3%), self-efficacy (3.2%), child behavior (2.8%), and then parental mental health (1.7%). We ran both the hierarchical regressions to determine whether socio-economic status variables, including, income, employment status, and education influenced the models for both facilitative and psychological controlling parenting. However, these variables did not contribute any variance to the models.

**Table 5:** Study 1: summary of hierarchical regression analysis for variables predicting facilitative parenting (FPS scores).

	B	*β*	95% CI	*t*	*sr*^2^	*R*^2^	*R*^2^(adj.)
**Step 1**						0.043	0.030
Age of mother (years)	0.01	0.22	[0.01, 0.02]	2.52^*^	0.041		
Age of child (years)	-0.01	-0.05	[-0.04, 0.02]	-0.55	0.002		
**Step 2**						0.179	0.162
Age of mother (years)	0.01	0.11	[-0.01, 0.02]	1.28	0.011		
Age of child (years)	0.01	0.03	[-0.02, 0.03]	0.36	0.000		
SDQ	-0.02	-0.34	[-0.03, -0.01]	-4.92^***^	0.136		
**Step 3**						0.260	0.235
Age of mother (years)	0.01	0.12	[-0.01, 0.02]	1.35	0.009		
Age of child (years)	0.01	0.03	[-0.02, 0.03]	0.41	0.001		
SDQ	-0.01	-0.25	[-0.02, -0.01]	-3.04^**^	0.048		
DASS-21	-0.01	-0.14	[-0.01, 0.00]	-1.76	0.016		
PSOC-Efficacy	-0.02	0.26	[0.01, 0.03]	3.39^**^	0.059		
**Step 4**						0.370	0.339
Age of mother (years)	0.00	0.07	[-0.01, 0.01]	0.85	0.003		
Age of child (years)	0.01	0.05	[-0.02, 0.03]	0.69	0.002		
SDQ	-0.01	-0.19	[-0.02, 0.00]	-2.49^*^	0.028		
DASS-21	-0.01	-0.15	[-0.01, 0.00]	-1.98^*^	0.017		
PSOC-Efficacy	-0.01	0.20	[0.01, 0.02]	2.70^**^	0.032		
Compassionate goals	0.16	0.28	[0.08, 0.25]	3.82^***^	0.065		
Self-image goals	-0.12	-0.27	[-0.18, -0.06]	-3.75^***^	0.063		

*n = 150. CI, confidence interval; SDQ, strengths and difficulties questionnaire; DASS-21, depression and anxiety stress scale-21; PSOC – Efficacy, parenting sense of competence – efficacy subscale. ^∗^p < 0.05. ^∗∗^p < 0.01. ^∗∗∗^p < 0.001.*

## Discussion

The results of our first study indicated that parental motive, specifically whether it is driven by compassionate or self-image goals, was associated with distinct parenting styles. Self-image goals predicted psychological controlling parenting, whereas compassionate goals predicted facilitative parenting. Although Study 1 provides insight into the importance of goal orientation in relation to parenting style, it does not allow for causal inferences. Thus, Study 2 will examine the link between goals and emotions in a parenting context using an experimental design.

## Study 2

The cross-sectional nature of the first study limits the capacity to draw causal inferences about the influence of compassionate and self-image goals in a parenting context. A second study was conducted to address this limitation by experimentally manipulating compassionate and self-image goals in an online questionnaire. This study was based on the previous experimental work of Breines and Chen (2012) who found that self-compassion can increase self-improvement motivation after experiencing a failure. In study 2 participants were randomized to either a compassionate, self-image, or control condition. Conditions were manipulated by varying the instructions provided to participants (adapted from Breines and Chen, 2012, Study 3). Participants then read about various difficult parenting scenarios and reported their emotional responses. The control condition was included to examine baseline emotional responses in the absence of any goal orientation stimuli.

It was hypothesized that those in the compassionate goal condition would experience more positive and less negative emotional responses compared to those in the self-image goal (H1) and control (H2) conditions. We also explored whether those in the self-image goal condition would experience more negative and less positive emotional responses compared to the control condition.

## Materials and Methods

### Design and Participants

The study used a between-groups experimental design, with a manipulated between-groups variable of goal orientation. Participants were randomly allocated to one of three conditions, (a) compassionate goal, (b) self-image goal, or (c) control. The dependent variables were self-reported emotions in response to parenting scenarios. Demographics were measured using the FBQ and psychological control was measured using the PPC to control for differences between groups in age of the mother and child and trait psychological control (see section “Study 1”).

The study was advertised in the same way as Study 1. An a priori power analysis using the software program G^∗^Power (Faul et al., 2007) indicated that 159 participants would be required to obtain adequate power (0.80) to detect a medium effect size of 0.25 at the standard 0.05 alpha error probability. A total of 270 respondents voluntarily accessed the online survey. Parents with a child aged 3–9 years were eligible to participate. Fifty-three participants were excluded (not a parent, *n* = 3; child not aged 3–9 years, *n* = 4; did not read instructions upon allocation to condition, *n* = 1; missing all data, *n* = 33; only provided demographic information, *n* = 12). There were 6 males (2.76%) and 211 females (97.2%). As in Study 1, males were removed from further analyses. A further 13 participants were excluded due to inadequate sampling, leaving a final sample of *N* = 198 mothers aged 21–55 years (*M* = 36.05 years, *SD* = 6.10) with a child aged 3–9 years (*M* = 5.43 years, *SD* = 2.02). Participants were randomly allocated to conditions, with 70 allocated to the compassion condition, 66 to self-image and 62 to the control. There was no significant difference in age of the mother, age of the child or psychological control across conditions (see Table 6).

**Table 6:** Study 2: participant demographic characteristics and trait psychological control according to condition.

	Compassion (*n* = 70)		Self-Image (*n* = 66)		Control (*n* = 62)		Difference between conditions	
**Demographics**	*M*	*SD*	*M*	*SD*	*M*	SD	*F* (2, 194)	*p*
Age of mother^a^ (*n* = 197)	36.01	6.21	35.62	6.12	36.55	6.01	0.37	0.692
	*M*	*SD*	*M*	*SD*	*M*	*SD*	*F* (2, 195)	*p*
Age of child	5.62	2.12	5.23	1.87	5.36	2.03	0.68	0.508
**Control measure**	*M*	*SD*	*M*	*SD*	*M*	*SD*	*F* (2, 195)	*p*
Psychological control	62.07	13.17	64.93	18.17	61.13	13.90	1.10	.336

*^a^One participant in the compassion condition did not disclose their age.*

### Measures

#### Demographic Information

The FBQ was used to collect demographic information (see Study 1).

#### Emotional Responses

Participants were asked to imagine their child in a variety of brief parenting scenarios, adapted from Kirby et al. (2019). There were six scenarios in total describing problematic behavior of the child. These scenarios included: (1) imagine your child having a tantrum in public; (2) imagine your child is not doing well at childcare/school; (3) imagine your child has been accused of bullying; (4) imagine your child doesn’t do what you ask them when in public; (5) imagine your child swears when in public; and (6) imagine your child hits another child and makes them cry when in public. Participants’ reactions to each parenting scenario was assessed using a subscale of emotions, and a subscale of reflect shame, that has been used previously in the Kirby et al. (2019) study.

#### Emotions

Participants’ emotional responses to the parenting scenarios were measured for seven different emotions adapted from Goetz et al. (2010), which was also used in the Kirby et al. (2019) study. Participants were asked to indicate what emotions they felt in terms of anxiety, stress, sadness, anger, frustration, calmness and sympathy. Participants responded on a 10-point Likert scale from 1 (*Not at all*) to 10 (*The most you could feel*). An average score was then calculated across all six scenarios for each emotion. Higher scores for each emotion indicated higher levels. Internal consistency for each emotion across the six scenarios was good (ranging from α = 0.75 to α = 0.87).

#### Reflected Shame

Three items that assessed reflected shame that was experienced in relation to the parenting scenarios, which was also used in the Kirby et al. (2019) study. Participants were asked *“To what extent would you worry that other people would”* (1) *See you as an incompetent parent*, (2) *Look down on you*, and (3) *See you as a bad parent* on a 7-point Likert scale from 1 (*Strongly Disagree*) to 7 (*Strongly Agree*). An average score was then calculated across all six scenarios for each reflected shame item. Higher scores indicated greater reflected shame. Internal consistency was good (ranging from α = 0.84 to α = 0.85).

#### Psychological Control

The PPC measure used in Study 1 was similarly used to measure trait psychological control. Internal consistency for Study 2 was excellent (α = 0.93).

### Procedure

A convenience sample was used involving snowball sampling via online social media, which meant a website link would be posted, which when clicked, would direct participants to take part in the experiment. Participants completed the experiment using the online survey software package Qualtrics^TM^. All the participants provided written online informed consent before the start of their participation.

After completing the demographic details, participants were then randomly assigned to one of three experimental conditions using the randomization function within Qualtrics: (a) compassionate goal, (b) self-image goal, or (c) control condition. Participants were then presented with a set of instructions that differed depending on condition, which contained the manipulation, adapted from Breines and Chen (2012), Study 3.

Those in the compassionate goal condition read*: “In this next section we want you to remember that parenting is hard. We all face challenges, setbacks and disappointments. You are not alone with this. Try not to be too hard on yourself. We all try our best. Please answer the next set of questions with this in mind.”* Those in the self-image condition read: *“In this next section, we want you to remember that parents try to avoid making mistakes so that they don’t look like a bad parent. We try to get our children to do things our way because we know what is best for them. We all try our best. Please answer the next set of questions with this in mind.”* Those in the control condition read: *“In this next section, please answer the questions as best as you can.”*

Following the instructions, participants were then presented with six parenting scenarios, which they were asked to read and then indicate their emotional responses to each scenario. Participants then completed a set of manipulation check questions, followed by the PPC. In total, the average time to complete the online experiment was 15 min.

#### Manipulation Checks

Three questions were included to assess whether participants fully engaged with the online study. The first question “*How well do you remember the instructions that you were provided with before responding to the parenting scenarios?*” was rated on a seven-point Likert scale from 1 (Extremely well) to 7 (Not well at all). Two questions “*Did you closely read the initial instructions prior to reading about parenting scenarios?*” and “*Did the instructions help you to feel compassionate when responding to the parenting scenarios?*” were rated on a seven-point Likert scale from 1 (very true) to 7 (untrue).

### Data Analysis Plan

Prior to conducting analyses, missing data and assumptions were examined. Preliminary analyses compared the three conditions (compassionate goal, self-image goal and control) on demographic items and psychological control using one-way between-groups analysis of variance (ANOVAs). Manipulation checks were also assessed.

For experimental analyses, one-way between-groups ANOVAs were conducted to test whether those in the compassionate goal condition would experience more positive and less negative emotional responses compared to those in the self-image goal and control conditions and whether those in the self-image goal condition would experience more negative and less positive emotional responses compared to the control condition. To control for type one errors, Bonferroni adjustment was used, with a threshold for statistical significance of α = 0.005, two tailed.

## Results

### Data Screening

#### Missing Data

Analyses were conducted to determine the pattern of missing data for the dependent variables, manipulation check items, and PPC items. All variables were adequately assessed with data obtained for more than 50% of participants. Missing Values Analysis revealed a non-significant Little’s MCAR test χ^2^ (457, *N* = 198) = 478.88, *p* = 0.231, indicating that the data were MCAR. Expectation Maximization was used to estimate missing data.

### Preliminary Analyses

#### Control Measures

##### Demographics

A one-way between-groups ANOVA was conducted to compare the three conditions in terms of age of the mother and child. There were no significant differences between groups, suggesting that the randomization process produced equally comparable groups (see Table 6).

##### Psychological control

A one-way between-groups ANOVA was conducted to compare conditions according to total PPC scores. There was no significant difference between groups, suggesting that the randomization process produced equally comparable groups (see Table 6).

##### Manipulation checks

One-way between-groups ANOVAs were conducted to assess manipulation checks. The first check assessed memory for condition instructions. There was no significant difference between the compassion (*M* = 2.59, *SD* = 1.65), self-image (*M* = 2.50, *SD* = 1.43), and control (*M* = 3.17, *SD* = 1.93) conditions in memory for the instructions, *F* (2, 195) = 3.02, *p* = 0.051. The second check assessed whether participants closely read the instructions. Results revealed a significant difference between conditions, *F* (2, 195) = 14.03, *p* < 0.001, η^2^ = 0.13. *Post hoc* comparisons using the Tukey HSD test indicated that participants in the control condition (*M* = 3.30, *SD* = 1.90) were less likely to have closely read the instructions compared to the compassion (*M* = 2.10, *SD* = 1.37) and self-image (*M* = 1.99, *SD* = 1.35) conditions. The third check assessed whether the manipulation increased compassion within the participant. Results revealed a significant difference between conditions *F* (2, 195) = 5.94, *p* = 0.003, η^2^ = 0.06, with a medium effect. *Post hoc* comparisons using the Tukey HSD test indicated that participants in the control condition (*M* = 4.49, *SD* = 1.75) felt less compassionate compared to the compassion (*M* = 3.77, *SD* = 1.75) and self-image (*M* = 3.49, *SD* = 1.55) conditions.

### Main Analyses

#### Emotional Responses

One-way between-groups ANOVAs were conducted to compare the effect of goal orientation (compassion, self-image and control) on participant’s emotional responses in terms of emotions and reflected shame in response to the brief parenting scenarios. There was no significant difference between conditions across the seven emotions items or the three reflected shame items, all *p* > 0.005. See Table 7 for a summary of all emotional responses.

**Table 7:** Study 2: one-way between-groups ANOVA results for dependent measure outcomes between compassion, self-image and control conditions.

Dependent variable	Compassion (*n* = 70)			Self-image (*n* = 66)			Control (*n* = 62)			Difference between conditions	
	*M*	*SD*	95% CI	*M*	*SD*	95% CI	*M*	*SD*	95% CI	*F*(2, 195)	*p*
**Emotions**											
Anxiety	5.57	2.13	[5.16, 6.18]	5.65	1.98	[5.16, 6.13]	5.66	1.67	[5.23, 6.08]	0.00	0.998
Stress	6.05	1.92	[5.59, 6.51]	5.85	1.81	[5.40, 6.29]	5.95	1.69	[5.52, 6.38]	0.22	0.806
Sadness	5.28	1.74	[4.87, 5.70]	5.16	1.74	[4.74, 5.58]	5.56	1.83	[5.10, 6.03]	0.87	.423
Anger	4.86	2.14	[4.36, 5.37]	5.00	1.78	[4.57, 5.44]	4.73	1.84	[4.25, 5.19]	0.33	0.718
Frustration	5.73	2.12	[5.23, 6.24]	5.81	1.63	[5.41, 6.21]	5.90	1.75	[5.46, 6.34]	0.13	0.876
Calm	3.65	1.79	[3.23, 4.08]	4.39	1.65	[3.99, 4.80]	3.61	1.69	[3.18, 4.04]	4.35	0.014
Sympathetic	4.26	1.36	[3.94, 4.59]	4.88	1.41	[4.53, 5.22]	4.36	1.65	[3.94, 4.78]	3.36	0.037
**Reflected shame**											
Incompetent	4.25	1.27	[3.95, 4.56]	4.43	1.21	[4.14, 4.73]	4.04	1.20	[3.73, 4.34]	1.63	0.198
Looked down on	4.30	1.30	[3.99, 4.61]	4.52	1.19	[4.22, 4.81]	4.02	1.19	[3.71, 4.32]	2.64	0.074
Bad parent	4.25	1.24	[3.77, 4.38]	4.45	1.17	[4.16, 4.74]	4.08	1.21	[3.77, 4.38]	1.49	0.227

*ANOVA, analysis of variance; CI, confidence interval. Alpha set to 0.005.*

#### Age of Child

We also conducted a series of ANOVAs to determine whether age of the child influenced the emotional response of the parent in our scenarios. We found no significant differences in the emotional responses reported by parents across child’s age.

## Discussion

Parents were randomly assigned to either a compassionate goal, self-image goal or control condition. Conditions were experimentally manipulated by varying the framing of instructions provided to participants. Inconsistent with all hypotheses, no differences in emotional responses were observed between conditions. Our view is the brief instructions provided to induce compassionate and self-image orientation were insufficient, and potentially a stronger intervention such as a meditation exercise (e.g., 10 min listening to audio guided exercise) might be more appropriate and helpful to tap into motivational shift. In sum, the findings of Study 2 suggest that emotional responses to difficult parenting scenarios do not differ according to whether participants were prompted with compassionate goal, self-image goal, or control instructions.

## General Discussion

Does a parent’s motivation matter when it comes to parenting? Our findings are somewhat mixed, but it would appear that parental motivation does at least have some impact on parental style. However, further experimental work is needed to determine how modifiable compassionate motivational shift can be with parents, and whether this changes emotional reactions to difficult parenting scenarios.

To the best of our knowledge, this is the first study that has specifically examined parental motivation and how it may predict parenting style. In support of our pre-registered hypotheses we found that that high self-image goals were uniquely associated with greater psychological control, after accounting for child behavior, parental mental health, and parental self-efficacy. Specifically, the results suggest that the more an individual had self-image goals, the more they reported the use of psychologically controlling parenting. This finding is consistent with previous research by Mills et al. (2007), and similarly indicates that self-focused concerns may lead to psychologically controlling parenting. As previously discussed, people with self-image goals are theorized to operate from an *egosystem* motivational perspective, which is characterized by prioritization of one’s own needs (Crocker et al., 2009) and construction of a public image that reflects the individual’s ideal self (Crocker and Canevello, 2008; Crocker et al., 2009). The finding that self-image goals predict psychological control is consistent with egosystem theory, in that those with self-image goals may employ psychological control strategies to control the child in order to meet their own needs, at the expense of the child’s development of an independent sense of self.

We also found support for our second hypotheses that high compassionate goals were uniquely associated with greater facilitative parenting, after accounting for child behavior, parental mental health, and parental self-efficacy. Specifically, the more an individual had compassionate goals, the more they reported the use of warm and responsive parenting behaviors, characterized by facilitative parenting. This finding is consistent with previous research showing that compassion is associated with responsiveness to the needs of others (Canevello and Crocker, 2011) and greater observed warmth in mothers during interaction with their child (Miller et al., 2015). Additionally, this result aligns with the theory that people with compassionate goals operate from an *ecosystem* motivational perspective, through an understanding of interconnectedness with others (Crocker et al., 2009). Specifically, those with compassionate goals are responsive to the needs of others, evidenced by the use of warm and responsive parenting strategies that support the child’s social skills and relationships.

Exploratory analysis revealed that high compassionate goals were related to reduced psychological control, and that high self-image goals were related to reduced facilitative parenting, after accounting for child behavior, parental mental health, and parental self-efficacy. This suggests that a compassionate goal orientation is associated with reduced psychologically controlling parenting, whereas a high self-image goal orientation is associa-ted with reduced facilitative parenting. Importantly, compassion-ate goals were found to have the strongest explanatory power in both psychological control and facilitative parenting.

Inconsistent with hypotheses, Study 2 found that those who were prompted to adopt compassionate goals did not experience more positive and less negative emotional responses compared to those in the self-image goal and control conditions, in response to reading about difficult parenting situations. Moreover, we found no age interactions, thus implying our scenarios seemed to work similarly with all ages. This finding is inconsistent with previous research showing that self-image goals are linked to anxiety and stress (Erickson et al., 2018) and that self-compassion is linked to more positive and less negative emotional responses in parents (Kirby and Baldwin, 2018). Moreover, the findings are inconsistent with previous research from which Study 2 was adapted, which showed that a subtle reminder to be self-compassionate following initial failure at a test lead to increased time spent studying for a subsequent test compared to those who read a self-esteem statement or those in the no intervention control condition (Breines and Chen, 2012, Study 3).

Although Study 2 was the first to experimentally manipulate goal orientation, we were unable to establish a causal link between goals and emotional responses to difficult parenting situations. We propose that there are at least three possible explanations for this. First, results revealed that the manipulation was unsuccessful, as those in the compassionate goal and self-image goal conditions felt equally compassionate compared to the control. Thus, it is possible that the brief instructions were insufficient to induce compassionate and self-image goal orientations as intended. This could be partly due to the instructions. That is, the self-image instructions could have inadvertently elicited aspects of self-compassion, particularly in relation to common humanity. The self-image instructions describes how all parents try to avoid making mistakes and try not to look like a bad parent. Instead of priming parents for self-image goals this may have elicited a sense of common humanity, as it indicated that we are not alone with our uncertainties and self-image worries but that we share these with other parents. Thus, the intended self-image goal prompt might actually be a self-compassionate prompt, specifically in relation to common humanity. This is supported by the manipulation checks that showed differences in feelings of compassion for those in control condition as compared to compassion condition as well as self-image condition (but not between compassion and self-image).

Second, Breines and Chen (2012) found the self-compassionate prompt was able to facilitate greater motivation in university students to pass an exam, this finding might not generalize to parents, where there is an interaction between two individuals (parent and child). In contrast to an instructional prompt that was used in our study, in a previous study we used a 15-min Loving-Kindness Meditation, where parents focused on sending intentions of good will to oneself, a person that made them smile (e.g., their child), a stranger, someone they disliked, and to a group of people (e.g., a family). In the Kirby and Baldwin (2018) study they found the 15-min meditation led to increased positive responses to the vignettes (e.g., calm and sympathetic) and less negative responses (e.g., frustration and anger) compared to a focused imagery group. We suggest, that for parents, a longer and more embodied intervention such as the LKM meditation is required to bring about emotional shift to stressful parenting situations. This suggestion is in line with recent findings from Matos et al. (2018) who found that brief compassionate mind training was effective in helping individuals with distress, but this was moderated by an individual’s capacity to embody compassion. Embodiment refers to an individual’s ability to bring and feel compassion into everyday life. For example in a stressful parenting situation slowing the breath and trying to think through, “*If I was at my compassionate wisest and strongest how would I like to think, how would I like to act, in this moment.*” We suggest future research should examine a parent’s capacity to embody compassionate motivation, as this could be a key aspect to facilitating shit to compassionate goals. Moreover, we adopted a between –groups design, a pre-post design with a longer intervention might be more appropriate to assess for motivational shift in parents (Kirby et al., 2019).

Third, the current study measured emotions adapted from Goetz et al. (2010), which were trait-based emotions. In contrast, Breines and Chen (2012), Study 3 assessed self-improvement behavior by measuring time spent studying for a test, which is a commonly used and objective and sensitive measure (Di Paula and Campbell, 2002; Williams and Desteno, 2008). Thus, the measurement of emotions may have been less sensitive, and the brief instructions may have been unable to override parents’ trait emotions.

### Implications for Compassion and Parenting Programs

The finding that a compassionate goal orientation was the strongest predictor of positive facilitative parenting, as well as lower levels of psychological controlling parenting, supports the growing call for parenting programs to consider integrating compassion-based approaches within their intervention design (Coatsworth et al., 2010; Kirby, 2017; Waters, 2017). Compassion Focused Therapy (Gilbert, 2014) was developed to cultivate compassionate motivation to help individuals who struggle with self-criticism and shame, with a growing evidence-base supporting its effectiveness (Kirby et al., 2017). Compassion Focused Therapy is theoretically informed by social mentality theory, which is consistent with Crocker and Canevello’s (2008) ego- and ecosystem model. However, given the lack of findings in Study 2 regarding the use of an instructional prompt to facilitate motivational shift in parents, further work is needed to determine what level of dosage is required to help facilitate change.

### Limitations and Directions for Future Research

Although the present research has provided a deeper insight parental motivation, there are a number of limitations that should be addressed. First, only a small proportion of males volunteered to participate in both studies. Thus, the decision was made to exclude males, limiting generalizability to fathers. By excluding fathers it meant we could exclusively examine the role of parental motives and shame proneness in mothers, and as the majority of parenting research has been conducted with mothers it allows for easier comparisons to past research (Nowak and Heinrichs, 2008). However, this is a limitation of our study and future research should actively recruit equivalent proportions of mothers and fathers. Moreover, research has shown differential effects between mothers and fathers, with an indirect association between shame proneness and psychological control, through a worrisome approach toward the child identified among fathers (Mills et al., 2007). Thus, it would also be important to compare compassionate and self-image goals in mothers and fathers in order to determine whether they operate similarly for both parents.

A further limitation of the current research is that recruitment relied on self-selection. People who volunteer their time for the purpose of psychological research tend to be highly conscientious (Lönnqvist et al., 2007) and have greater intellectual ability, interest and motivation compared to non-volunteers (Rosenthal, 1965). Future research could include an incentive for participation to encourage participation from a more representative sample to minimize bias and increase generalizability of results. Another limitation is that both studies relied on self-report measures. Thus, participants’ responses may not reflect how parents actually behave with their children, limiting generalizability of results to parents’ behavior. Moreover, although the parenting scenarios used in Study 2 have been used previously (Kirby et al., 2019), they were hypothetical scenarios and may not reflect the parents child’s behavior. Current research relies heavily on introspection and hypothetical responses, thus the need for behavioral observation has been suggested (Baumeister et al., 2007). Future research in the form of an observational study could further inform our understanding of the influence of compassionate and self-image goals on parenting. This could involve measuring compassionate and self-image goals in parents and providing parents with difficult tasks to complete with their child, such as the challenging puzzle and origami tasks used by Miller et al. (2015). As in the study conducted by Miller et al. (2015), warmth (e.g., praise, encouragement, and hugs) and negativity (e.g., criticism, aggravated tone, and disapproval) could similarly be observed and coded to examine the link between goals and positive and aversive parenting behaviors. It could be anticipated that those with high compassionate goals would display greater warmth and reduced negativity compared to those with high self-image goals.

Finally, in relation to Study 2, given trait compassion and self-esteem have been found to be strongly associated with self-image goals and compassionate-goals, we could have included assessment measures to control for this to exclude the possibility that dispositional characteristics would differ between the groups.

## Conclusion

The present research is the first to examine compassionate and self-image goals in parents. The findings suggest that in addition to child behavior, parental mental health, and self-efficacy, a high self-image goal orientation is linked to increased psychologically controlling parenting, whereas a high compassionate goal orientation is linked to greater facilitative parenting. Our findings suggest instructional prompts for motivational shift are unsuccessful, and possibly stronger interventions are required when attempting to shift parents from self-image to compassionate motives It is recommended that further experimental work is conducted that attempts to cultivate compassionate motivation in parents to determine whether this can influence change in parental style and child outcomes.

## Ethics Statement

This study was carried out in accordance with the recommendations of The University of Queensland Ethics Board Committee with informed consent collected from all subjects. All subjects gave informed consent in accordance with the Declaration of Helsinki. The protocol was approved by The University of Queensland Ethics Board Committee.

## Author Contributions

JK came up with the original idea of study one and assisted OG with data analysis and writing. OG helped to design study two and executed both study one and study two, and assisted with the data analyses, and helped to writing the manuscript. PG collaborated in the writing and editing of the final manuscript. All authors approved the final version of the manuscript for submission.

## Conflict of Interest Statement

The authors declare that the research was conducted in the absence of any commercial or financial relationships that could be construed as a potential conflict of interest.

## Footnotes

1 https://osf.io/8vc73/ and https://osf.io/p268g/

## References

[B1] Abidin R. R. (1992). The determinants of parenting behavior. *J. Clin. Child Psychol.* 21 407–412. 10.1207/s15374424jccp210412

[B2] Anthony L. G. Anthony B. J. Glanville D. N. Naiman D. Q. Waanders C. Shaffer S. (2005). The relationships between parenting stress, parenting behaviour and preschoolers’ social competence and behaviour problems in the classroom. *Infant Child Dev.* 14 133–154. 10.1002/icd.385

[B3] Antony M. M. Bieling P. J. Cox B. J. Enns M. W. Swinson R. P. (1998). Psychometric properties of the 42-item and 21-item versions of the depression anxiety stress scales in clinical groups and a community sample. *Psychol. Assess.* 10 176–181. 10.1037/1040-3590.10.2.176

[B4] Barber B. K. (1996). Parental psychological control: revisiting a neglected construct. *Child Dev.* 67 3296–3319. 10.1111/j.1467-8624.1996.tb01915.x 9071782

[B5] Barber B. K. Harmon E. L. (2002). “Violating the self: parental psychological control of children and adolescents,” in *Intrusive Parenting: How Psychological Control Affects Children and Adolescents*, ed. Barber B. K. (Washington, DC: American Psychological Association), 15–52.

[B6] Baumeister R. F. Vohs K. D. Funder D. C. (2007). Psychology as the science of self-reports and finger movements: whatever happened to actual behavior? *Perspect. Psychol. Sci.* 2 396–403. 10.1111/j.1745-6916.2007.00051.x 26151975

[B7] Belsky J. de Haan M. (2011). Annual research review: parenting and children’s brain development: the end of the beginning. *J. Child Psychol. Psychiatry* 52 409–428. 10.1111/j.1469-7610.2010.02281.x 20626527

[B8] Breines J. G. Chen S. (2012). Self-compassion increases self-improvement motivation. *Personal. Soc. Psychol. Bull.* 38 1133–1143. 10.1177/0146167212445599 22645164

[B9] Canevello A. Crocker J. (2011). Interpersonal goals, others’ regard for the self, and self-esteem: the paradoxical consequences of self-image and compassionate goals. *Eur. J. Soc. Psychol.* 41 422–434. 10.1002/ejsp.808

[B10] Canevello A. Crocker J. (2017). Compassionate goals and affect in social situations. *Motiv. Emot.* 41 158–179. 10.1007/s11031-016-9599-x 28890583 PMC5584879

[B11] Cecil C. A. M. Barker E. D. Jaffee S. R. Viding E. (2012). Association between maladaptive parenting and child self-control over time: cross-lagged study using a monozygotic twin difference design. *Br. J. Psychiatry* 201 291–297. 10.1192/bjp.bp.111.107581 22918964

[B12] Coatsworth J. D. Duncan L. G. Greenberg M. T. Nix R. L. (2010). Changing parent’s mindfulness, child management skills and relationship quality with their youth: results from a randomized pilot intervention trial. *J. Child Fam. Stud.* 19 203–217. 10.1007/s10826-009-9304-8 24013587 PMC3765025

[B13] Cowan C. S. M. Callaghan B. L. Kan J. M. Richardson R. (2016). The lasting impact of early-life adversity on individuals and their descendants: potential mechanisms and hope for intervention. *Genes Brain Behav.* 15 155–168. 10.1111/gbb.12263 26482536

[B14] Crocker J. Canevello A. (2008). Creating and undermining social support in communal relationships: the role of compassionate and self-image goals. *J. Pers. Soc. Psychol.* 95 555–575. 10.1037/0022-3514.95.3.555 18729694

[B15] Crocker J. Canevello A. (2011). “Egosystem and ecosystem motivational perspectives on caregiving,” in *Moving Beyond Self-interest: Perspectives from Evolutionary Biology, Neuroscience, and the Social Sciences*. eds Brown S. L. Brown R. M. Penner L. A. (Oxford: Oxford University Press).

[B16] Crocker J. Olivier M.-A. Nuer N. (2009). Self-image goals and compassionate goals: costs and benefits. *Self Ident.* 8 251–269. 10.1080/15298860802505160 21218194 PMC3017354

[B17] Di Paula A. Campbell J. D. (2002). Self-esteem and persistence in the face of failure. *J. Pers. Soc. Psychol.* 83 711–724. 10.1037/0022-3514.83.3.711 12219864

[B18] Eisenberg N. Fabes R. A. Schaller M. Carlo G. Miller P. A. (1991). The relations of parental characteristics and practices to children’s vicarious emotional responding. *Child Dev.* 62 1393–1408. 10.1111/j.1467-8624.1991.tb01613.x 1786723

[B19] Erickson T. M. Granillo M. T. Crocker J. Abelson J. L. Reas H. E. Quach C. M. (2018). Compassionate and self-image goals as interpersonal maintenance factors in clinical depression and anxiety. *J. Clin. Psychol.* 74 608–625. 10.1002/jclp.22524 28898407

[B20] Faul F. Erdfelder E. Lang A.-G. Buchner A. (2007). G^∗^Power 3: a flexible statistical power analysis program for the social, behavioral, and biomedical sciences. *Behav. Res. Methods* 39 175–191. 10.3758/BF03193146 17695343

[B21] Gilbert P. (2014). The origins and nature of compassion focused therapy. *Br. J. Clin. Psychol.* 53 6–41. 10.1111/bjc.12043 24588760

[B22] Goetz J. L. Keltner D. Simon-Thomas E. (2010). Compassion: an evolutionary analysis and empirical review. *Psychol. Bull.* 136 351–374. 10.1037/a0018807 20438142 PMC2864937

[B23] Goodman R. (1997). The strengths and difficulties questionnaire: a research note. *J. Child Psychol. Psychiatry* 38 581–586. 10.1111/j.1469-7610.1997.tb01545.x 9255702

[B24] Hart C. H. Newell L. D. Olsen S. F. (2003). “Parenting skills and social-communicative competence in childhood,” in *Handbook of Communicationand Social Interaction Skills*, eds Greene J. O. Burleson B. R. (Mahwah, NJ: Lawrence Erlbaum Associates), 753–797.

[B25] Healy K. L. Sanders M. R. Iyer A. (2015a). Facilitative parenting and children’s social, emotional and behavioral adjustment. *J. Child Fam. Stud.* 24 1762–1779. 10.1007/s10826-014-9980-x

[B26] Healy K. L. Sanders M. R. Iyer A. (2015b). Parenting practices, children’s peer relationships and being bullied at school. *J. Child Fam. Stud.* 24 127–140. 10.1007/s10826-013-9820-4

[B27] Henry J. D. Crawford J. R. (2005). The short-form version of the depression anxiety stress scales (DASS-21): construct validity and normative data in a large non-clinical sample. *Br. J. Clin. Psychol.* 44 227–239. 10.1348/014466505X29657 16004657

[B28] Jackson A. P. (2000). Maternal self-efficacy and children’s influence on stress and parenting among single black mothers in poverty. *J. Fam. Issues* 21 3–16. 10.1177/019251300021001001

[B29] Johnston C. Mash E. J. (1989). A measure of parenting satisfaction and efficacy. *J. Clin. Child Psychol.* 18 167–175. 10.1207/s15374424jccp18028

[B30] Kirby J. N. (2016). The role of mindfulness and compassion in enhancing nurturing family environments. *Clin. Psychol.* 23 142–157. 10.1111/p.12149

[B31] Kirby J. N. (2017). “Compassion-focused parenting,” in *The Oxford Handbook of Compassion Science*, eds Seppälä E. M. Simon-Thomas E. Brown S. L. Worline M. C. Cameron D. Doty J. R. (Oxford: Oxford University Press), 1–27.

[B32] Kirby J. N. Baldwin S. (2018). A randomized micro-trial of a loving-kindness meditation to help parents respond to difficult child behavior vignettes. *J. Child Fam. Stud.* 27 1614–1628. 10.1007/s10826-017-0989-9

[B33] Kirby J. N. Sampson H. Day J. Hayes A. Gilbert P. (2019). Human evolition and culture in relationship to shame in the parenting role: implications for psychology and psychotherapy. *Psychol. Psychother.* 92 238–260. 10.1111/papt.12223 30932312

[B34] Kirby J. N. Tellegen C. L. Steindl S. R. (2017). A meta-analysis of compassion-based interventions: current state of knowledge and future directions. *Behav. Ther.* 48 778–792. 10.1016/j.beth.2017.06.003 29029675

[B35] Koren-Karie N. Oppenheim D. Dolev S. Sher E. Etzion-Carasso A. (2002). Mothers’ insightfulness regarding their infants’ internal experience: relations with maternal sensitivity and infant attachment. *Dev. Psychol.* 38 534–542. 10.1037/0012-1649.38.4.534 12090483

[B36] Laurin J. C. Joussemet M. Tremblay R. E. Boivin M. (2015). Early forms of controlling parenting and the development of childhood anxiety. *J. Child Fam. Stud.* 24 3279–3292. 10.1007/s10826-015-0131-9

[B37] Lönnqvist J. E. Paunonen S. Verkasalo M. Leikas S. Tuulio-Henriksson A. Lönnqvist J. (2007). Personality characteristics of research volunteers. *Eur. J. Personal.* 21 1017–1030. 10.1002/per.655

[B38] Lovibond S. H. Lovibond P. F. (1995). *Manual for the Depression Anxiety Stress Scales*, 2nd Edn. Sydney: Psychology Foundation of Australia.

[B39] Mash E. J. Johnston C. (1990). Determinants of parenting stress: illustrations from families of hyperactive children and families of physically abused children. *J. Clin. Child Psychol.* 19 313–328. 10.1207/s15374424jccp19043

[B40] Matos M. Duarte J. Duarte C. Gilbert P. Pinto-Gouveia J. (2018). How one experiences and embodies compassionate mind training influences its effectiveness. *Mindfulness* 9 1224–1235. 10.1007/s12671-017-0864-1

[B41] McDowell D. J. Parke R. D. Wang S. J. (2003). Differences between mothers’ and fathers’ advice-giving style and content: relations with social competence and psychological functioning behavior in middle childhood. *Merrill-Palmer Q.* 49 55–76. 10.1353/mpq.2003.0004

[B42] Medvedev O. N. Krägeloh C. U. Titkova E. A. Siegert R. J. (2019). Rasch analysis and ordinal-to-interval conversion tables for the depression, anxiety and stress scale. *J. Health Psychol.* 10.1177/1359105318755261 [Epub ahead of print]. 29402132

[B43] Mikulincer M. Shaver P. R. (2016). “An attachment perspective on compassion and prosocial virtues,” in *Compassion: Concepts, Research, and Applications*, ed. Gilbert P. (Abingdon: Routledge).

[B44] Miller J. G. Kahle S. Lopez M. Hastings P. D. (2015). Compassionate love buffers stress-reactive mothers from fight-or-flight parenting. *Dev. Psychol.* 51 36–43. 10.1037/a0038236 25329554 PMC4351818

[B45] Mills R. S. L. Freeman W. S. Clara I. P. Elgar F. J. Walling B. R. Mak L. (2007). Parent proneness to shame and the use of psychological control. *J. Child Fam. Stud.* 16 359–374. 10.1007/s10826-006-9091-4 10800017

[B46] Nowak C. Heinrichs N. (2008). A comprehensive meta-analysis of triple P-Positive parenting program using hierarchical linear modeling: effectiveness and moderating variables. *Clin. Child Fam. Psychol. Rev.* 11 114–144. 10.1007/s10567-008-0033-0 18509758

[B47] Odgers C. L. Caspi A. Russell M. A. Sampson R. J. Arseneault L. Moffitt T. E. (2012). Supportive parenting mediates neighborhood socioeconomic disparities in children’s antisocial behavior from ages 5 to 12. *Dev. Psychopathol.* 24 705–721. 10.1017/S0954579412000326 22781850 PMC3551477

[B48] Ohan J. L. Leung D. W. Johnston C. (2000). The parenting sense of competence scale: evidence of a stable factor structure and validity. *Can. J. Behav. Sci.* 32 251–261. 10.1037/h0087122

[B49] Olsen S. F. Yang C. Hart C. H. Robinson C. C. Wu P. Nelson D. A. (2002). “Maternal psychological control and preschool children’s behavioral outcomes in China, Russia, and the United States,” in *Intrusive Parenting: How Psychological Control Affects Children and Adolescents*, ed. Barber B. K. (Washington, DC: American Psychological).

[B50] Perkins S. C. Finegood E. D. Swain J. E. (2013). Poverty and language development: roles of parenting and stress. *Innov. clin. Neurosci.* 10 10–19. PMC3659033 23696954

[B51] Raphael-Leff J. (1986). Facilitators and regulators: conscious and unconscious processes in pregnancy and early motherhood. *Br. J. Med. Psychol.* 59 43–55. 3964585 10.1111/j.2044-8341.1986.tb02664.x

[B52] Robinson C. C. Mandleco B. Olsen S. F. Hart C. H. (1995). Authoritative, authoritarian, and permissive parenting practices: development of a new measure. *Psychol. Rep.* 77 819–830. 10.1007/s10995-016-2145-3 27469108

[B53] Rodgers A. Y. (1998). Multiple sources of stress and parenting behavior. *Child. Youth Serv. Rev.* 20 525–546. 10.1016/S0190-7409(98)00022-X

[B54] Rosenthal R. (1965). The volunteer subject. *Hum. Relat.* 18 389–406. 10.1177/001872676501800407

[B55] Sanders M. R. Kirby J. N. (2014). A public-health approach to improving parenting and promoting children’s well-being. *Child Dev. Perspect.* 8 250–257. 10.1111/cdep.12086

[B56] Sanders M. R. Kirby J. N. Tellegen C. L. Day J. J. (2014). The Triple P-Positive Parenting program: a systematic review and meta-analysis of a multi-level system of parenting support. *Clin. Psychol. Rev.* 34 337–357. 10.1016/j.cpr.2014.04.003 24842549

[B57] Sanders M. R. Mazzucchelli T. G. (2018). “How parenting influences the lives of children,” in *The Power of Positive Parenting: Transforming the Lives of Children, Parents, and Communities Using The Triple P System*. (1st ed.), eds Sanders M. R. Mazzucchelli T. G. (New York, NY: Oxford University Press).

[B58] Sanders M. R. Woolley M. L. (2005). The relationship between maternal self-efficacy and parenting practices: implications for parent training. *Child Care Health Dev.* 31 65–73. 10.1111/j.1365-2214.2005.00487.x 15658967

[B59] Waters L. (2017). *The Strength Swtich.* New York, NY: Penguin Random House.

[B60] Williams L. A. Desteno D. (2008). Pride and perseverance: the motivational role of pride. *J. Pers. Soc. Psychol.* 94 1007–1017. 10.1037/0022-3514.94.6.1007 18505314

[B61] Zubrick S. R. Silburn S. R. Garton A. F. Burton P. Dalby R. Carlton J. (1995). *Western Australian Child Health Survey: Developing Health and Well-being in the Nineties.* Perth, WA: Australian Bureau of Statistics.

